# An NS-3 Implementation and Experimental Performance Analysis of IEEE 802.15.6 Standard under Different Deployment Scenarios

**DOI:** 10.3390/ijerph17114007

**Published:** 2020-06-04

**Authors:** Beom-Su Kim, Tae-Eung Sung, Ki-Il Kim

**Affiliations:** 1 Department of Computer Science and Engineering, Chungnam National University, Daejeon 34134, Korea; bumsou10@cnu.ac.kr; 2 Department of Computer and Telecommunications Engineering, Yonsei University, Wonju 26493, Korea; tesung@yonsei.ac.kr

**Keywords:** wireless body area networks (WBAN), IEEE 802.15.6, NS-3 implementation

## Abstract

Various simulation studies for wireless body area networks (WBANs) based on the IEEE 802.15.6 standard have recently been carried out. However, most of these studies have applied a simplified model without using any major components specific to IEEE 802.15.6, such as connection-oriented link allocations, inter-WBAN interference mitigation, or a two-hop star topology extension. Thus, such deficiencies can lead to an inaccurate performance analysis. To solve these problems, in this study, we conducted a comprehensive review of the major components of the IEEE 802.15.6 standard and herein present modeling strategies for implementing IEEE 802.15.6 MAC on an NS-3 simulator. In addition, we configured realistic network scenarios for a performance evaluation in terms of throughput, average delay, and power consumption. The simulation results prove that our simulation system provides acceptable levels of performance for various types of medical applications, and can support the latest research topics regarding the dynamic resource allocation, inter-WBAN interference mitigation, and intra-WBAN routing.

## 1. Introduction

IEEE 802.15.6 [1] is a new wireless communication standard for wireless body area networks (WBANs). WBANs are mainly used for monitoring physical conditions, such as the heart rate and blood pressure, for recording electrocardiograms (ECGs) and electroencephalograms (EEGs), and for forwarding physical data to a hub. The purpose of IEEE 802.15.6 is to provide an international standard for a short-range, low power, and reliable communication in or around the human body. This contrasts with other approaches, such as low-rate wireless personal area networks (WPANs), based on IEEE 802.15.4 [2], which require more power and bandwidth. Thus, research into WBANs must thoroughly comply with the IEEE 802.15.6 standard to meet its strict requirements and regulations.

The latest research topics based on the IEEE 802.15.6 standard can be classified into three categories. The first is a study on dynamic resource allocation or transmission scheduling scheme for intra-WBAN communications. Technically, designing such a intra-WBAN resource allocation or transmission scheduling scheme is quite challenging due to the following reasons: (i) unpredictable movements of the human body and changes in posture can significantly degrade the network performance; and (ii) available resources are very limited and the battery cannot be replaced because the medical sensors are attached in or on the human body; and (iii) significant amounts of RF energy (i.e., specific absorption rate (SAR) is the rate at which the human body absorbs radio frequency (RF) radiation) can increase the body temperature and cause tissue damage. For example, if a channel is assigned to a node that is likely to fail transmission, then resources will be wasted. On the other hand, excessive resource allocation biased to a specific node shortens network life and damages body tissue. That is, the resource allocation or transmission scheduling policy must consider effects on portable antennas, radiation pattern, and changes in characteristics as a result of the body movement. The second is a study on channel sharing mechanism between adjacent BANs. As we enter an aging society, the data transmissions in the small area (e.g., a hospital) will be explosively increased in the near future. However, the existing channel sharing mechanisms can hardly meet the requirements for e-health services in a wireless hospital environment. The third is the study of intra-WBAN routing protocol. Although the WBAN topology is basically based on a single-hop star topology, a number of routing schemes using body movements are also being studied to optimize the transmission power or prevent the temperature rise of the human body.

As we described above, there are critical research challenges that need to be solved before the e-health monitoring system is applied to real life. To meet the aim of achieving a reliable medical system, these restrictions should be addressed in advance through appropriate simulations. However, existing IEEE 802.15.6 simulation systems cannot support the aforementioned research topics because they provide a simplified network model without any major components specified in the IEEE 802.15.6 standard such as connection-oriented resource allocations, inter-WBAN interference mitigation, or a two-hop star topology extension. As a result, the performance verification methods used in recent studies are somewhat questionable, a common problem being the omission of the frame exchange procedure of the IEEE 802.15.6 MAC protocol (e.g., a MATLAB-based simulation), or non-compliance with the MAC/PHY parameters defined in the IEEE 802.15.6 standard. For example, current WBAN routing protocols [3,4,5] do not follow the IEEE 802.15.6 MAC standard for a performance verification, or merely describe the concept of the proposed protocol. That is, they ignore the frame exchange sequence for a BAN configuration, transmission scheduling, and resource allocation. As a result, it is unlikely that their mechanism can be applied to real medical systems. In addition, current IEEE 802.15.6 MAC studies [6,7,8] have evaluated their mechanisms based on the IEEE 802.15.4 standard. That is, the simulation results are unreliable unless some mechanisms for a resource allocation, channel usage agreement, inter-WBAN interference mitigation, and two-hop star topology extension specified in the IEEE 802.15.6 standard are reflected in their protocol design. In conclusion, their performance verification methods cannot meet the medical constraints or communication regulations for certain medical applications.

To meet the requirements of various medical applications and improve the reliability of the simulation results, numerous simulation platforms for WBANs have been proposed. First, IEEE 802.15.4 based WBAN simulation platforms have been proposed [9,10,11,12]. Their prototype systems are based on a beacon mode with superframes. The superframe structure is divided into a contention access period (CAP) and a contention free period (CFP). The authors constructed a single BAN consisting of a hub and several sensor nodes. To support a heterogeneous traffic flow, the hub can reserve one or more time slots for a time-critical data application. The non-time-critical data are transmitted to the hub using CSMA/CA. Although the IEEE 802.15.4 based simulation systems provide the useful functions necessary for node-to-hub communication, they cannot meet the specific requirements presented in the IEEE 802.15.6 standard. For example, to provide a differentiated quality-of-service (QoS), the IEEE 802.15.6 standard classifies traffic types into eight levels according to the user priorities, and uses the values assigned to each level as a back-off counter for CSMA/CA. In addition, the IEEE 802.15.6 standard provides complementary mechanisms to mitigate channel interference with neighboring BANs, such as beacon shifting, channel hopping, and active superframe interleaving, although most of them do not support these features.

To overcome the limitations of IEEE 802.15.4-based WBAN simulation platforms, more realistic WBAN simulation systems have been proposed [13,14,15,16,17,18,19]. In the simulation system, a WBAN consists of heterogeneous nodes with different requirements. The authors modified the CSMA/CA algorithm based on the IEEE 802.15.6 standard to provide a user priority service. To build a more realistic simulation system, they designed the PHY model according to the frequency band supported by the IEEE 802.15.6 standard and measured the performance using the modified CSMA/CA. In addition, numerical formulas were derived to evaluate the performance of the proposed system in terms of its energy consumption, throughput, and delay. However, other access modes, such as a beacon mode with superframes and a non-beacon mode without superframes, are not provided, and thus it is difficult to verify the accurate performance under different WBAN scenarios. Moreover, realistic simulations cannot be conducted because the channel model or propagation loss model applied to the simulation system ignores the body shadowing or channel fading caused by human mobility.

To reflect non-ideal on-body channel characteristics for use in the simulation system, in [20,21,22,23], a simulation system is proposed for modeling an on-body channel and evaluating the performance of WBAN transmission schemes. The authors defined a new propagation loss model and calculated the specific reception sensitivity based on the position of the node pair. The proposed on-body channel models allow the simulation system to conduct a more realistic performance evaluation under various WBAN scenarios. However, most of the scenarios define an on-body channel model for a single WBAN without considering the channel interference caused by the surrounding environment. In other words, in a public area consisting of numerous local WBANs, such as a hospital, the WBAN system should provide an appropriate coexistence mechanism between neighboring WBANs. However, the proposed systems do not provide a channel interference mitigation mechanism between adjacent WBANs. In addition, they do not provide a set of communication functions required for a realistic WBAN simulation (e.g., a connection request frame or connection assignment frame exchange process for the association between a hub and a node).

In summary, existing WBAN simulation systems cannot be used to conduct a realistic WBAN simulation under different network scenarios, nor do they support the current research trends in WBANs because they do not consider the following components specified in the IEEE 802.15.6 standard. First, to send higher priority data, the IEEE 802.15.6 MAC provides a priority-based random-access phase and managed access phase (MAP) in beacon mode with superframes. MAP is used to arrange unscheduled allocation intervals, scheduled allocation intervals, and improvised polling access intervals, and is necessary to support the QoS of emergency data or time critical data. In addition, MAP plays an important role in adaptive resource management in response to body movements. However, most WBAN simulation systems provide only a random-access phase for a frame transmission. Second, the existing simulation systems try to eliminate intra-WBAN interference using CSMA/CA. Further, having smooth coexistence mechanisms with neighboring BANs is important to avoid inter-WBAN interference. That is, a WBAN simulation system should provide a complementary mechanism to mitigate channel interference with neighboring WBANs. Third, existing simulation systems assume a one-hop star topology for a network consisting of several sensor nodes connected to a hub. In a one-hop star topology, the data frames between a hub and node are exchanged directly. However, to evaluate the routing protocols studied in WBANs, such as temperature-aware [24], energy-aware [25], and mobility-aware routing [26], an additional complementary mechanism (i.e., two-hop star topology extension) should be provided in the simulation system.

To make the WBAN simulation platform closer to a practical WBAN system and support the current research trends regarding WBANs, in this study, we review the major components of the IEEE 802.15.6 standard and present modeling strategies for implementing the IEEE 802.15.6 MAC on an NS-3 simulator. In addition, we configure various simulation scenarios, such as a heterogeneous traffic flow, channel sharing between multi-WBANs, and a two-hop star network topology for a realistic performance evaluation.

We can achieve the following benefits by accurately implementing the components specified in the IEEE 802.15.6 standard; (i) the accurate performance of the proposed system model can be evaluated in advance before we develop the actual WBAN prototype; and (ii) it is possible to determine whether the proposed system model can meet the strict requirements of various medical applications (i.e., scalability) or whether it can be applied to actual medical systems (i.e., adaptability); and (iii) by providing a prototype for a WBAN, it is possible to design a specific system model with the new modern technologies (e.g., blockchain, machine learning).

The main contributions of this paper are summarized below.

- We provide a comprehensive review of the major components of the IEEE 802.15.6 standard and propose modeling strategies for implementing the IEEE 802.15.6 MAC on an NS-3 simulator. The proposed modeling strategies can also be useful when researchers build a wireless personal area network system based on an NS-3 simulator.
- Unlike existing simulation systems for WBANs, we implement core mechanisms such as an association, connection-oriented link allocation, channel hopping mechanism, and a two-hop star topology extension. By using these mechanisms, our simulation system can support the latest research topics regarding the dynamic resource allocation/transmission scheduling, inter-WBAN interference mitigation, and intra-WBAN routing.
- To meet the aim of achieving a reliable health monitoring system, we model the necessary components on an NS-3 simulator, including the PHY, MAC, error rate, propagation loss, on-body channel, energy, and human mobility models. In addition, we present specific network configurations required for a realistic simulation. By providing a realistic simulation environment, it is possible to determine whether the proposed WBAN model can meet the strict requirements of medical applications.
- We evaluate the performance of the proposed simulation system under realistic WBAN scenarios such as a heterogeneous traffic flow, channel sharing between multi-WBANs, and two-hop star network topology. Such network scenarios make our simulation platform closer to that of a practical WBAN system and allow us to pre-determine whether the proposed WBAN system can be applied to the real world before we develop the actual medical system.
- The IEEE 802.15.6 standard has already been optimized for a WBAN system, although there are additional constraints that need be considered in the MAC protocol design. We present future studies that should be reflected in the WBAN system and introduce the lastest WBAN research domains where the proposed simulation system can be used.

The remainder of this paper is organized as follows. Section 2 describes previous related studies. In Section 3, the major components of IEEE 802.15.6 MAC are described. In Section 4, we describe the design strategies of IEEE 802.15.6 MAC on an NS-3 simulator. A performance evaluation is then detailed in Section 6. Areas of future study and some concluding remarks are finally presented in Section 7 and Section 8, respectively.

## 2. Related Studies

To build a reliable health monitoring system, a WBAN communication technology needs to consider not only the requirements of various medical applications, but also efficient resource management schemes and bio-effects that reflect the characteristics of the human body. Thus, these restrictions need to be addressed in advance through appropriate simulations. As shown in Table 1, there are various simulation platforms that provide a realistic WBAN simulation environment as well as useful frameworks such as OPNET, OMNET++, CANet, MATLAB and NS-3, however, most of them do not provide a specific prototype for a WBAN. In other words, if there are major components not supported by the simulation system, we cannot perform an accurate performance evaluation. For example, if the simulation system does not support a two-hop star topology extension, all intra-routing protocols cannot be simulated because the IEEE 802.15.6 MAC standard basically considers one-hop transmission. Similarly, the resource allocation/transmission scheduling policy cannot be customized unless the simulation system supports the association mechanism (i.e., connection-oriented link allocation) between a hub and a node. In this section, we discuss the limitations of previous WBAN studies using the existing WBAN simulation systems.

Since the IEEE 802.15.6 standard was first released, numerous researchers have adopted strategies to modify or extend the IEEE 802.15.4 system and provide a BAN-specific simulation environment. C. Li et al. [9] conducted WBAN simulations based on an IEEE 802.15.4 system modeled in standard C. They constructed a single BAN consisting of a hub and sensor nodes. The proposed simulation system uses beacon mode with superframes specified in the IEEE 802.15.4 standard to support heterogeneous WBAN traffic. The superframe structure is divided into CAP and CFP. The hub reserves one or more time slots in CFP for a time-critical data transmission. CAP is used for a non-time-critical data transmission. The same approach is used in [10]. In addition to applying the same superframe structure, their system provides a scheduled polling access phase using the OMNET++ and MiXim framework. M. Deylami et al. [11] provided an IEEE 802.15.4-based WBAN simulation system designed to mitigate the harmful effects of the coexistence between neighboring BANs. They modeled the WBAN system using the IEEE 802.15.4 framework provided by the OPNET network modeler. In the proposed system, each BAN should exchange control messages with other local BANs to synchronize the order of the channel usage. Y. Kim et al. [12] proposed an IEEE 802.15.4-based WBAN simulation system for building a health monitoring system in a WBAN/WiFi coexistence environment, and developed a new discrete event-driven simulator to evaluate their adaptive load control algorithm. Their system follows the superframe structure of the IEEE 802.15.4 standard.

Although an IEEE 802.15.4-based WBAN simulation system can cover the major issues of a body area network, it has difficulty meeting the different requirements of medical applications such as the throughput, delay, and power consumption. To provide a user-priority service according to the medical application, as an example, the IEEE 802.15.6 standard classifies traffic types into eight levels according to the user priorities and uses the values assigned to each priority level as a back-off counter for CSMA/CA. In addition, the resource allocation schemes and complementary mechanisms specified in the IEEE 802.15.6 standard are not supported.

To overcome the limitations of the existing IEEE 802.15.4-based WBAN simulation platforms, numerous WBAN-specific simulation systems based on the IEEE 802.15.6 standard have been proposed. To verify the performance of a WBAN-specific simulation system, S. Ullah et al. [13,27] constructed a WBAN system using standard C++. Their system measures the performance under maximum throughput and an average delay over the different frequency bands and data rates supported by the IEEE 802.15.6 physical layer. However, the proposed system assumes that the channel condition is perfect and that the bit error rate (BER) is zero. In addition, it provides only a priority-based contention access phase for a frame transmission. J. Y. Khan et al. [14] presented a specific WBAN design technique for medical applications. They also reviewed the design issues of the WBAN MAC protocol required for reducing the energy consumption. They then constructed a WBAN system based on the CSMA/CA architecture and analyzed the performance of the patient monitoring applications using the OPNET network modeler. However, their proposed system also assumes ideal channel conditions and does not provide a flexible resource allocation scheme. L. Z. Kahsay et al. [15] constructed a WBAN-specific communication system using a user priority-based random back-off scheme in a wireless hospital environment. The simulation system is built using the OPNET network modeler. However, the proposed system offers limited communication functions and frameworks, which makes it difficult to evaluate the performance under various WBAN environments. H. Fourati et al. [16] proposed a WBAN system for the CANet e-health project. The CANet (Cane Network) project aims to implement a monitoring system for the elderly that can be used in everyday life. Their system follows the superframe structure of the IEEE 802.15.6 standard and provides a user priority-based random-access phase (RAP). However, the superframe structure does not include scheduled access phases or polling access phases specified in the IEEE 802.15.6 standard. In addition, W. Yue et al. [17] constructed a WBAN simulation platform on an NS-3 simulator. Their system configures a specific WBAN simulation scenario according to the practical application. In addition, they modify or implement some modules, such as a CSMA/CA module, PHY module, and MAC module provided by the NS-3, based on the IEEE 802.15.6 standard. However, the proposed system provides only a random-access phase for the frame transmission and omits the functions necessary for resource management such as a connection-oriented link allocation. The same approach is used in [18], but similarly, the proposed prototype system provides only basic communication functions. That is, the proposed WBAN systems are designed for a single BAN, and thus complementary mechanisms such as channel interference mitigation with adjacent BANs are not supported. X. Yuan et al. [19] constructed coexisting WBAN environments and evaluated the normalized throughput and average delay between coexisting WBANs based on the IEEE 802.15.6 CSMA/CA mechanism. However, their system does not implement channel-sharing mechanisms such as beacon shifting, channel hopping, or active superframe interleaving specified in the IEEE 802.15.6 standard. Finally, some research work such as [28] and introduced in [29] have been conducted to evaluate performance through MATLAB.

In summary, the existing WBAN-specific simulation systems commonly aim to provide differentiated user priority services according to medical applications. To achieve this goal, most of them modify the CSMA/CA algorithm based on the user priorities. However, they do not provide other access modes, such as the beacon mode with superframes or non-beacon mode without superframes, and thus it is difficult to verify the accuracy of the performance under different WBAN scenarios. In addition, the IEEE 802.15.6 standard provides complementary mechanisms to mitigate channel interference with neighboring BANs, such as beacon shifting, channel hopping, and active superframe interleaving, but most of them do not support these features. Moreover, the proposed WBAN simulation systems assume a one-hop star topology for a network consisting of several sensor nodes connected to a hub. That is, a two-hop star topology extension mode should be provided in the simulation system to support the routing protocols being studied in the WBANs [24,25,26]. Moreover, realistic simulations cannot be conducted because the channel model or propagation loss model applied to their simulation systems ignores the body shadowing or channel fading caused by human mobility.

To consider non-ideal on-body channel characteristics, WBAN-specific channel models and frameworks have been proposed [20,21,22,23]. In these studies, WBAN simulation systems are proposed for modeling an on-body channel and evaluating the performance of WBAN transmission schemes. Their systems define a new propagation loss model and an on-body channel model based on the position of the node pair. However, most of them define the channel models for a single BAN without considering the channel interference caused by the surrounding environment. In addition, they also do not provide various access phases required for realistic simulations such as scheduled access or improvised polling access.

To deal with the aforementioned problems, we review the major components of the IEEE 802.15.6 standard and present modeling strategies for implementing the IEEE 802.15.6 MAC on an NS-3 simulator. In addition, we configure various simulation scenarios, such as a heterogeneous traffic flow, channel sharing between multi-WBANs, and two-hop star network topology for a realistic performance evaluation. The major components of the IEEE 802.15.6 MAC layer are described in the following section.

## 3. Major Components of IEEE 802.15.6 MAC Layer

The IEEE 802.15.6 standard provides significant flexibility to users by providing unique mechanisms such as association, access mode, access phase, acknowledgment policy, two-hop star topology extension, and inter-WBAN interference mitigation. A network simulator based on the IEEE 802.15.6 standard must implement the following components.

### 3.1. Association

A node sends a non-command management type frame to the hub to be assigned a one-octet node identifier (NID) from the hub, and the hub responds with an NID (0x02-0xF5) in the recipient ID field of the ACK message. After receiving an authorized NID from the hub, the node may receive or transmit a message from a node or hub belonging to the same BAN. An association is a series of processes used for constructing a local body area network between a hub and nodes and is essential for preventing inter-WBAN interference and communication with unauthorized nodes.

### 3.2. Access Mode

**Figure 1:**
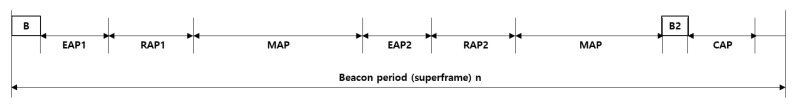
Beacon mode with superframes.

**Figure 2:**
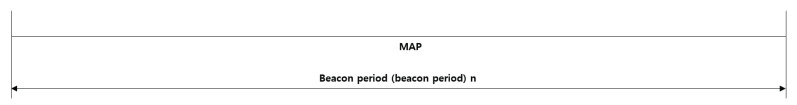
Non-beacon mode with superframes.

**Figure 3:**
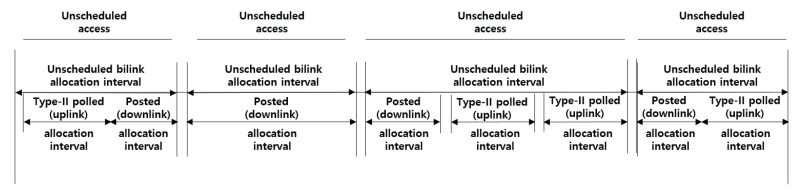
Non-beacon mode without superframes.

A hub should choose an appropriate access mode, based on the channel conditions, application requirements, coexistence consideration, and policy regulations to reduce the risk of mutual interference with other electronic devices or to save resources. As shown in Figure 1, Figure 2 and Figure 3, a hub operates in one of three access modes; beacon mode with superframes, non-beacon mode with superframes, and non-beacon mode without superframes. A hub operates in beacon mode, transmitting a beacon during every beacon period (superframes), to enable time referenced allocations. By contrast, a hub operates in non-beacon mode with superframes if access to the medium in its BAN involves time referencing, or without superframes if access to the medium in its BAN involves no time referencing.

### 3.3. Access Phase

**Figure 4:**
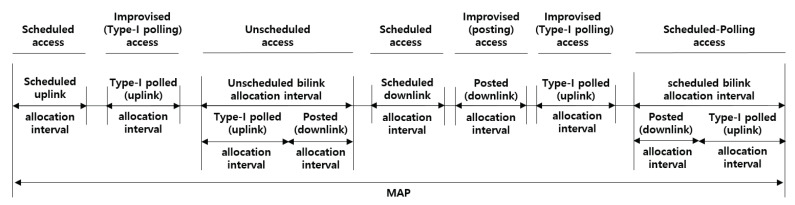
Allocation intervals and access methods in a managed access phase (MAP).

A hub organizes applicable access phases in each superframe, as illustrated in Figure 1. The hub should place the access phases, i.e., exclusive access phase (EAP), random access phase (RAP), contention access phase (CAP), and MAP, and apply scheduled, unscheduled, and improvised access options, as illustrated in Figure 4.

- **Random access**: The nodes that collect critical body data (e.g., EEG and ECG) take priority over those that collect general body data (e.g., blood pressure and pulse oximeter) in a channel contention. A node initiates frame transactions in EAP, RAP, and CAP using CSMA/CA. The contention window (CW) boundary of the CSMA/CA is determined through a predefined value between $CW_{min}$ and $CW_{max}$, which depends on different user priorities (UPs). The UPs for the CSMA/CA are divided into eight levels (0–7), giving a higher value to the node collecting critical body data.
- **Improvised access (connectionless contention-free access)**: In beacon or non-beacon mode with superframes, a hub can use improvised access to send polls and posts to a node without advance notice. The improvised access phase is only operated in MAP, as shown in Figure 4. A hub can employ improvised access as an independent access method to send polls or posts without a reservation or allocation made in advance, as a supplemental access method to send additional polls and posts outside scheduled/unscheduled allocations, or as an enabling access method for scheduled-polling access and unscheduled access to send polls or posts inside scheduled/unscheduled bi-link allocations.
- **Unscheduled access (connection-oriented contention-free access)**: A hub can use unscheduled access to obtain unscheduled bi-link allocations and polled and posted allocations therein (improvised access). To obtain a new unscheduled bi-link allocation, a node should send a connection request frame to the hub (i.e., association request). The node includes a Type-I unscheduled bi-link request (if its hub is operating on a beacon or non-beacon with superframes) or Type-II unscheduled bi-link request (if its hub is operating on non-beacon mode without superframes) in the frame header. To grant an unscheduled bi-link allocation requested by the node, a hub sends a connection assignment frame to the node, including a Type-I unscheduled bi-link assignment or Type-II unscheduled bi-link assignment. In an unscheduled bi-link allocation interval, the hub sends a pole or T-poll frame to the node to grant improvised uplink access, or transmits a control frame to the node through a downlink.
- **Scheduled access and scheduled-polling access (connection-oriented contention-free access)**: In beacon or non-beacon mode with superframes, a node and a hub can use the scheduled access to obtain scheduled uplink allocations (guaranteed uplink time slots) and scheduled downlink allocations (guaranteed downlink time slots), and can use the scheduled-polling access to obtain scheduled bi-link allocations and a polled and posted allocation therein (improvised access). A hub can arrange scheduled uplink allocation intervals, scheduled downlink allocation intervals, and scheduled bi-link allocation intervals. To obtain a new scheduled allocation, a node should send a connection request frame to the hub (i.e., an association request). The node includes an uplink request (if needed), a downlink request (if needed), and a bi-link request (if needed) in the frame header. To grant scheduled allocations requested by the node, a hub sends a connection assignment frame to the node, including an uplink assignment, a downlink assignment, and a bi-link assignment if each allocation is granted.

### 3.4. Acknowledgment Policy

The IEEE 802.15.6 MAC supports several ACK modes for saving resources and achieving scheduling efficiency. A node or hub can set the ACK policy field of a control type frame. A recipient can acknowledge a received frame by sending an immediate acknowledgment (I-ACK) or block acknowledgment (B-ACK) frame. Instead of I-ACK, the recipient can send an I-ACK+Poll or B-ACK+Poll frame to grant an immediate polled allocation or announce a future poll or post.

### 3.5. Two-Hop Star Topology Extension

**Figure 5:**
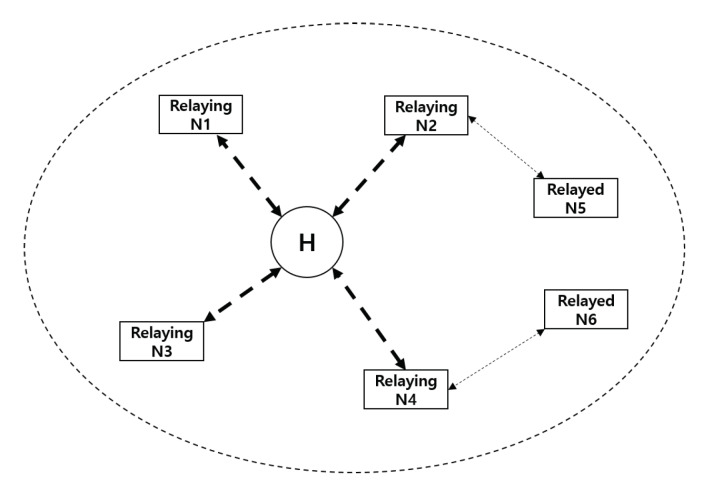
Two-hop star network topology.

Nodes can use a two-hop extension to forward frames through other nodes that can communicate directly with the hub, as shown in Figure 5. The relaying node or target hub can initiate a two-hop extension at the time determined by the initiator as appropriate. The relaying node can also send its own frame to the hub directly, as in a one-hop star topology. To implement various intra-body routing protocols (e.g., temperature-aware routing [24], energy-aware routing [25], and mobility-aware routing protocols [26]), the relayed and relaying nodes must both support a two-hop star topology extension.

### 3.6. Inter-WBAN Interference Mitigation

Each local BAN operates within the same frequency bands, which results in considerable interference. A hub can use one of the optional mechanisms for interference mitigation between its BAN and neighbor BANs as follows:

- **Beacon shifting**: A hub can transmit its beacons at different time offsets by including a beacon shifting sequence field in its beacon frame header.
- **Channel hopping**: A hub can change its operating channel in the operating frequency band periodically by including the channel hopping state and next channel hop fields in its beacon frame header.
- **Active superframe interleaving**: A BAN can share the same operating channel with other BANs with or without interleaving their active superframes.

## 4. Design of IEEE 802.15.6 MAC on NS-3

We refer to the interaction procedure between components of the IEEE 802.15.4 standard to construct the IEEE 802.15.6 prototype system. The MAC sublayer provides two services, accessed through two service access points (SAPs). All data frames and acknowledgment frames are processed in the MAC common part sublayer (MCPS). The MAC management services are processed in the MAC layer management entity (MLME). These two sublayers provide an interface between the next higher layer and the PHY through the PHY data sublayer (PD) and PHY layer management entity (PLME) interface.

### 4.1. MLME-SAP

**Figure 6:**
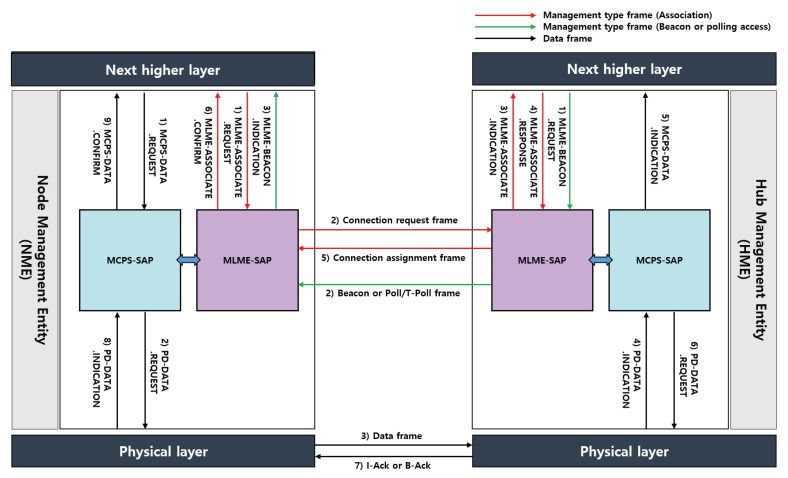
Design of IEEE 802.15.6 MAC on NS-3.

The major functions provided by the MLME-SAP are as follows:

- **Association and connection-oriented link allocation**: There are two ways to associate a node with a hub. The first method is an implicit association. An unassociated node sends a non-command management type frame with the sender ID field of the MAC header set to the unconnected_NID to the hub, and the hub responds with an NID in the recipient ID field of the I-Ack frame. The second method is an explicit association. An unassociated node explicitly transmits a connection request frame to the hub to connect with another hub. The proposed system supports an explicit association, as illustrated in Figure 6. The MLME-ASSOCIATE.REQUEST is generated by the next higher layer of an unassociated node and then forwarded to the MLME-SAP to request an association with a hub. After receiving the MLME-ASSOCIATE.REQUEST, the MLME-SAP creates a connection request frame with the sender ID field of the MAC header set to the unconnected_NID and sends it to the hub. In addition, in beacon or non-beacon mode with superframes, the connection request frame is used for requesting unscheduled/scheduled link allocations (connection-oriented contention-free access) as well as their association with a hub. If the MLME-SAP of a node successfully sends a connection request frame to the hub, the MLME-SAP of the hub generates an MLME-ASSOCIATE.INDICATION and forwards it to the next higher layer to indicate the reception of a connection request frame. The hub determines whether to accept or reject the unassociated node, and the next higher layer of the hub forwards the MLME-ASSOCIATE.RESPONSE to the MLME-SAP. After receiving the MLME-ASSOCIATE.RESPONSE from its next higher layer, the MLME-SAP transmits a connection assignment frame with the recipient ID field of the MAC header set to a confirmed NID to the node. In addition, if the connection request frame includes unscheduled/scheduled link allocation information requested by the node, the hub grants the requested link allocations through the connection assignment frame.
- **Setting up access phases and enabling optional mechanisms**: In an active superframe, a hub transmits a beacon and provides random access phases and improvised/unscheduled/scheduled access phases. The MLME-BEACON.REQUEST is generated by the next-higher layer of a hub and is issued to the MLME-SAP, as illustrated in Figure 6. We can enable the coexistence field of the beacon frame header, and beacon shifting, channel hopping, and superframe interleaving options are available. We can also enable the two-hop extension field of the beacon frame header using the MAC capability field. The improvised type-I, but not type-II, immediate polled allocation intervals and posted allocation intervals are obtained through a poll or T-poll frame in MAP. If the MLME-SAP of a hub successfully transmits a beacon or poll/T-poll frame, the MLME-SAP of a node generates the MLME-BEACON.INDICATION and forwards it to the next higher layer to indicate the reception of a beacon or poll/T-poll frame.
- **Operating channel selection and channel hopping**: The connection assignment frame generated by MLME-SAP of a hub includes a channel order information environment (IE) field and delivers an operating channel sequence for channel hopping through the channel bit map field of the corresponding IE. In addition, a hub can change its operating channel in the operating frequency band by including the channel hopping state and next channel hop fields in the beacon frame header. The operating channels may be determined by a hub through an appropriate channel selection algorithm (e.g., link quality-aware channel selection).

### 4.2. MCPS-SAP

**Figure 7:**
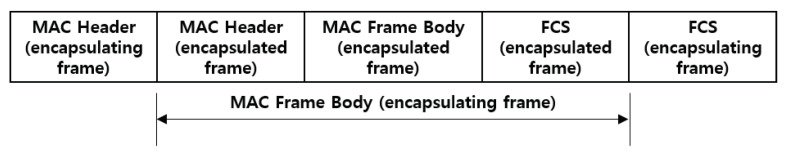
General frame encapsulation format.

The major functions provided by MCPS-SAP are as follows:

- **MAC data service**: The MCPS-SAP supports the transport of application data between a node and a hub. As illustrated in Figure 6, the application data are transferred in the form of an MCPS-DATA.REQUEST, and is generated by the next-higher layer. After receiving an MCPS-DATA.REQUEST, the MCPS-SAP builds a MAC protocol data unit (MPDU) and forwards it to the PD-SAP in the form of a PD-DATA.REQUEST. The PD-SAP converts the received MPDU into a PHY protocol data unit (PPDU) and transmits it to the target hub. The MCPS-DATA.INDICATION indicates the transfer of application data from the MCPS-SAP to the next-higher layer. If the application data are successfully transmitted, and an acknowledgment (I-Ack or B-Ack) is also received, the MCPS-SAP forwards the MCPS-DATA.CONFIRM to the next-higher layer.
- **Two-hop star topology extension**: A hub (i.e., target hub) and node (i.e., relayed node) can use a two-hop extension mode to exchange the management or data type frames through another node (i.e., a relaying node) connected with both of them. To send a management or data type frame, the relayed node sends an encapsulating “X” frame to the relaying node, as shown in Figure 7, wherein the recipient ID field of the MAC header is set to the NID of the relaying node, and the relay field of the MAC header is set to 1. The other fields of the MAC header are set as if the relaying node is a hub. Upon receiving an encapsulating “X” frame from the relayed node, the relaying node processes the encapsulating “X” frame with the following additional tasks: The sender ID of the MAC header is set to the NID of the relayed node, and the recipient ID of the MAC header is set to the ID of the target hub; in addition, the MAC frame body is encapsulated again as an encapsulating "X" frame. To do so, the relayed node must maintain a list of one-hop neighbor nodes.

## 5. MAP Scheduling Procedure on NS-3

**Figure 8:**
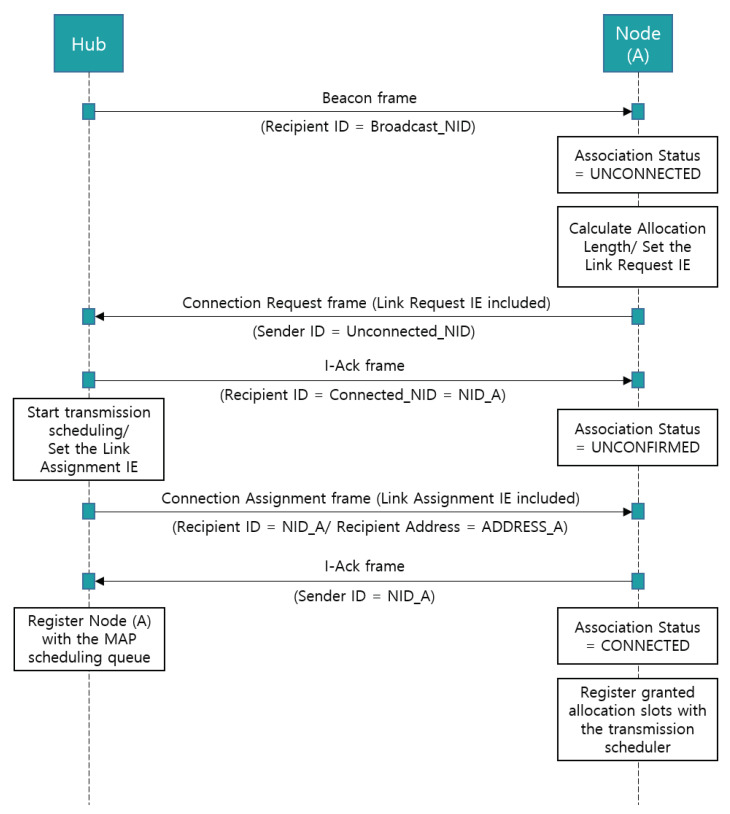
Association procedure.

To obtain scheduled/unscheduled allocation intervals (i.e., connection-oriented link allocation intervals), an association between a node and a hub should be established. Figure 8 shows the time-sequence diagram for the association. First, an unconnected node (A) initiates frame transactions during random access periods using CSMA/CA after receiving the beacon frame sent from the hub.

**Figure 9:**
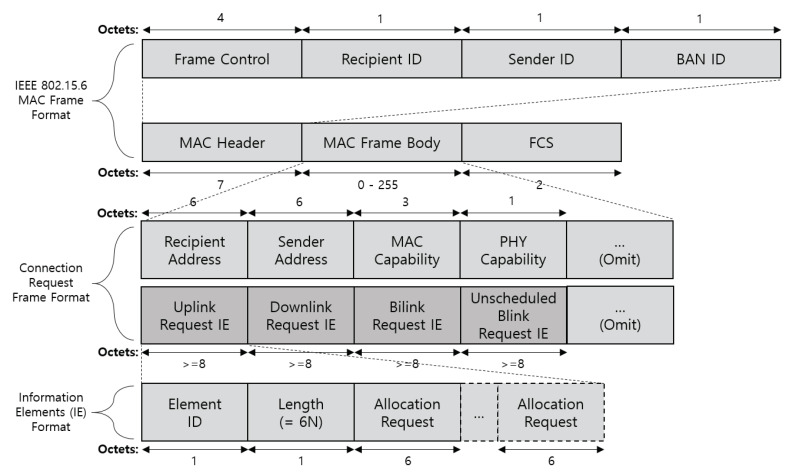
IEEE 802.15.6 MAC frame format and connection request frame format.

**Figure 10:**
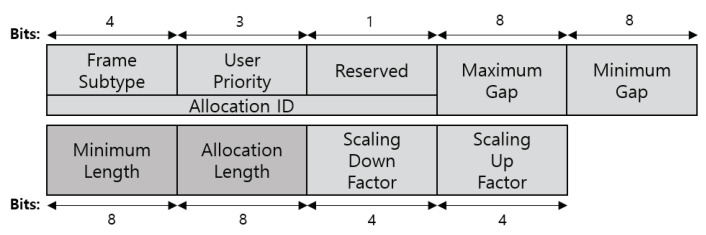
Allocation request format.

As shown in Figure 9, the connection request frame includes four types of link request IE fields. The link request IE consists of the element ID, length, and allocation request fields. The element ID field is set to a value that identifies the type of link request. The length field is set to the total length of the allocation request. The allocation request field is formatted as shown in Figure 10. The uplink request IE, downlink request IE, bi-link request IE, and unscheduled bi-link request IIE (Type-I) use the same allocation request format. The node needs to add allocation request information in the link request IE field (one or more allocation requests can be included) to request the link allocation interval of the MAP. The allocation ID identifies an allocation requested by the node. This field is used by the hub for UP-based link allocation. The maximum gap and minimum gap fields are set to the largest or smallest length between the end of an allocation interval and the start of the next allocation interval of the requested allocation. The minimum length and allocation length fields are set to the smallest length or overall length of the requested allocation (in units of allocation slots). The scaling down and scaling up factors are set to the number of allocation slots, which can be extended by several times. These fields should be determined by considering the network environment such as the channel condition and interference.

After calculating the required allocation length in each allocation interval, the node (A) sends a connection request frame with the sender ID field of the MAC header set to the unconnected_NID to the hub, and the hub responds with a connected_NID (NID_A) in the recipient ID field of the I-Ack frame. After receiving an authorized NID (NID_A) through the I-Ack frame, node (A) changes its association status to UNCONFIRMED until the corresponding connection assignment frame arrives.

To grant the requested link allocation and complete the association, the hub needs to send a connection assignment frame including the link assignment IE (the connection assignment frame format and link assignment IE format are defined in Sections 5.3.7 and 5.7.7 of the IEEE 802.15.6 standard, respectively). To set the link assignment IE of the connection assignment frame, the hub applies transmission scheduling for each allocation interval according to the link request IE of the connection request frame. Note that, the transmission scheduling is basically conducted on a first-come, first-serve basis.

However, designing such a transmission scheduling mechanism is quite challenging due to unpredictable movements of the human body. Moreover, some medical applications that collect vital body data are time-sensitive and delay-sensitive, so the user priority should be reflected in the scheduling policy. Note that, the dynamic transmission scheduling approaches are described in Section 7. The current version of the proposed simulation system preferentially allocates the scheduled uplink allocation intervals to nodes with higher UPs (i.e., $UP_{4}$ to $UP_{7}$) in the MAP. In addition, a hub employs improvised access as an independent access method to send polls or posts without a reservation allocation made in advance, as a supplemental access method. As a result, nodes with higher UPs can be assigned guaranteed time slots or have more opportunities to send data to the hub through improvised polling access. The impact of MAP scheduling on performance can be found in Section 6. After finishing the transmission scheduling, the hub registers a node (A) with the MAP scheduling queue and sends a connection assignment frame with the recipient ID field of the MAC header set to the NID_A. Note that, the MAP scheduling queue is used to unicast a poll/post frame to the connected node with a higher UP (i.e., $UP_{4}$ to $UP_{7}$). The connection assignment frame includes the link assignment IE that has an allocation interval start field and allocation interval end field. The interval start field indicates the start point of a scheduled uplink interval approved by the hub in the MAP (in units of allocation slots), and the interval end field indicates the end point of the allocation interval. If a node has not been granted an allocation interval, the corresponding fields are initialized to 255 and 0, respectively. Upon receiving the connection assignment frame from the hub, node (A) responds with the I-Ack frame and changes its association status to CONNECTED. Next, node (A) registers granted allocation slots with the transmission scheduler. The node wakes up at the interval start time by the scheduler and transmits its own data to the hub. Except for the granted allocation intervals (i.e., scheduled uplink/downlink allocation intervals), the transceiver state is switched to IDLE.

## 6. Performance Evaluation

The description of our validation study is presented in this section. First, the NS-3 models used for the simulation are described. The implementation of the model consists of a set of C++ classes. Next, specific simulator configurations are presented. Finally, each of the following subsections provides simulation scenarios required for verification. Each subsection describes the objective of the scenario first, followed by a discussion of the verification results.

### 6.1. Model Description

The NS-3 network simulator supports the physical layer and MAC layer models for configuring a low rate wireless personal area network (LR-WPAN). Basically, we use the network models provided by NS-3 to build a simulation environment for WBANs. However, the existing models only define simple frame transaction procedures or basic interactions between network layers. In addition, the PHY/MAC parameters defined in the LR-WPAN models are different from those defined in the IEEE 802.15.6 standard. Thus, to build a WBAN environment, the existing models should be modified or new models added. In this section, we provide LR-WPAN based modification strategies for implementing the IEEE 802.15.6 standard.

#### 6.1.1. Physical Layer

The IEEE 802.15.6 standard supports three operational PHYs: narrowband (NB), ultra-wideband (UWB), and human body communications (HBC). The hub employs a slotted ALOHA or CSMA/CA protocol depending on the selected PHY. In general, the CSMA/CA protocol is employed in NB PHY, and thus we use NB PHY in the simulation to implement a user-priority based CSMA/CA.

Table 2 summarizes the supported frequency bands and transmission parameters of the NB PHY [30]. However, because these parameters are requirements for transmitting radio signals in an actual WBAN environment, it is not necessary to implement the specifications in the simulation. The major functions of the IEEE 802.15.6 PHYs supported by an NS-3 simulator are a clear channel assessment (CCA), activation/deactivation of the radio transceiver, and transmission/reception of the physical layer protocol data unit (PPDU). The physical layer of the NS-3 consists of a PHY model, an error rate model, and a loss model. We model the PHY service specifications, PPDU formats, PHY constants, and protocol information base (PIB) attributes described in the IEEE 802.15.6 standard. The PIB to be managed in the PHY model are as follows: radio frequency channels, available channels supported by a channel page, transmit power, current channel page, maximum number of symbols in a frame, duration of the synchronization header in the symbols, and number of symbols per octet. The following functions are some of the important lists that the PHY model should implement.

- **PHY::SetTxPowerSpectralDensity**: Set the power spectral density of the outgoing signals.
- **PHY::SetNoisePowerSpectralDensity**: Set the noise power spectral density. The noise power density assumes a uniformly distributed thermal noise across the frequency bands.
- **PHY::SetChannel**: Registers a wireless channel model to be used in the PHY model.
- **PHY::ChannelSupported**: Check if the given channel is supported by the PHY model.
- **PHY::PhyIsBusy**: Check if the PHY is busy, which is the case if the PHY is currently sending or receiving a frame.
- **PHY::PdDataRequest**: Send a data frame to the wireless channel.
- **PHY::PlmeDataRequest**: Send a management frame to the wireless channel.
- **PHY::SetPdDataIndicationCallback**: Set the callback for the end of an RX, as a part of the interconnections between the PD-SAP and MCPS-SAP.
- **PHY::SetPlmeDataIndicationCallback**: Set the callback for the end of an RX, as a part of the interconnections between the PLME-SAP and MLME-SAP.
- **PHY::SetPlmeSetTRXStateConfirmCallback**: Set the callback for the end of an SetTRXState, as part of the interconnections between the PLME-SAP and MLME-SAP.
- **PHY::SetPlmeGetAttributeConfirmCallback**: Set the callback for the end of an GetAttribute, as part of the interconnections between the PLME-SAP and MLME-SAP.
- **PHY::SetPlmeCcaConfirmCallback**: Set the callback for the end of a CCA, as a part of the interconnections between the PHY and MAC. The CCA reports a busy medium upon detecting any energy above the receiver energy detection (ED) threshold.
- **PHY::SetPlmeEdConfirmCallback**: Set the callback for the end of an ED, as part of the interconnections between the PHY and MAC. The ED estimates the received signal power within the bandwidth of the channel. This callback is intended for use by upper layers for various tasks, including part of a channel hopping algorithm.

The radio model of the NS-3 simulator assumes a flat channel frequency response. Although the modulation scheme for each frequency is not used, the error model for each frequency band can be applied using the following functions; **PHY::SetPhyOption** and **PHY::SetErrorModel**. The error model description can be found in Section 8 of the IEEE 802.15.6 standard.

The PHY model uses the existing single spectrum channel model by registering the **SpectrumChannel** class provided by the NS-3. The propagation loss model can be registered using the following two functions: **SpectrumChannel::AddPropagationLossModel**, **SpectrumChannel::SetPropagationDelayModel**. The propagation model should set the RX power (dBm) based on the receiver sensitivity. These propagation models are defined in the propagation-module file. The loss model can fully utilize all existing simple loss models supported by the NS-3 simulator. However, the loss model should calculate the received power based on the position of the node pair and the radio power of the transmitter. In other words, the propagation loss model applied to the on-body channel model should consider the body shadowing and human mobility. For example, channel fading may occur between radio transmitters attached to the shoulder and leg according to the body posture. To apply these body characteristics to the existing propagation loss model, we set the average channel gain (dB), as shown in Table 3 [31].

After the RX power is estimated by the propagation loss model, the receiver calculates the signal-to-interference plus noise ratio (SINR) using the noise model registered through the **PHY::NoisePowerSpectralDensity** function. Finally, the PHY model can calculate the bit error rate (BER) and packet error rate (PER) using the SINR and RX power. The BER between the transmitter *i* and receiver *j* is calculated based on the SINR at time *t* and the transmission parameters of the PHY (e.g., modulation scheme and symbol rate) as follows [31]:

(1) $$BER_{ij}^{t}=\left\{\begin{array}{c} 0.5*e^{-Eb/No}\\ Ǫ(\sqrt{4^{Eb/No}}*\sin\left(\sqrt{2}*\pi /4\right)) \end{array}\right.$$

where Eb/No is the energy per bit-to-noise power spectral density ratio in dBm, which is calculated as follows [31]:

(2) $$Eb/No\left[\mathrm{dB}\right]=SINR_{ij}^{t}\left[\mathrm{dB}\right]+10*log_{10}\left(BW/R\right)$$

where BW denotes the bandwidth and *R* denotes the symbol rate. Finally, the PER is calculated using the following [31]:

(3) $$PER_{ij}=1-{\left(1-BER_{ij}^{t}\right)}^{n}$$

where *n* denotes the frame length in bits.

#### 6.1.2. MAC Layer

The MAC model is conceptually divided into MCPS-SAP and MLME-SAP, whereas the related functions are defined in the same space, i.e., the **WbanMac** class. The **WbanMac** class provides the IEEE 802.15.6 MAC functions, and manages various MAC state variables such as an association, two-hop extension, ACK mode, and channel hopping. The **WbanMac** class refers to separate classes for random access and time slotted access. The random access algorithm refers to the **LrWpanCsmaCa** class provided by an NS-3 simulator, and we change the CW values according to the user priorities, as shown in Table 4. Each node uniformly selects a random value from the interval (1, CW) as a back-off counter.

The following functions are some of the important lists that the MAC model should implement. Note that, to use the functions provided by the PHY and MAC classes, a separate helper class must be implemented. We implemented a helper class (**WbanHelper**) that provides interface functions such as an association, spectrum channel registration, mobility model registration, and network device management.

- **MAC::McpsDataRequest**: Request to transfer a MSDU.
- **MAC::SetMcpsDataIndicationCallback**: Set the callback for the indication of an incoming data frame. This callback implements MCPS-DATA.indication.
- **MAC::SetMcpsDataConfirmCallback**: Set the callback for confirmation of a data frame transmission request. This callback implements MCPS-DATA.confirm.
- **MAC::PdDataIndication**: Indicate the transfer of an MPDU from PHY to MAC.
- **MAC::PdDataConfirm**: Confirm the end of the transmission of an MPDU to MAC.
- **MAC::PlmeSetTRXState**: Set the PHY state (RX_ON, TRX_OFF, FORCE_TRX_OFF, TX_ON).
- **MAC::SetWbanMacState**: A random access algorithm (e.g., CSMA/CA) calls back the MAC after executing a channel assessment. MacState indicates a BUSY or IDLE channel condition.
- **MAC::SetAccessModeStatus**: Set the current access mode.
- **MAC::SetAssociationStatus**: Set the current association status.
- **MAC::SetTwoHopExtensionStatus**: Set the current topology extension status.
- **MAC::SetChannelHoppingStatus**: Set the current channel hopping status.
- **MAC::SendAck**: Send an acknowledgment packet (I-Ack or B-Ack) for the given sequence number.

#### 6.1.3. Energy Model

Most network simulators focus on modeling the wireless energy consumption because they assume that wireless communication consumes the most amount of power. The radio energy consumption model allows users to set the specific power consumption of the radio for different operating states. However, an NS-3 simulator does not provide any radio energy consumption model. To track the power consumption of the physical layer, we provide an energy consumption model. The **WbanEnergyModel** class estimates the energy consumption based on transceiver states such as TX, RX, and IDLE in the physical layer. The amount of power consumed in each state is shown in Table 5. The proposed energy model detects the changes in the state of the transceiver through the function that switches the state of the transceiver (i.e., MAC::PlmeSetTRXState). The node waits in the IDLE state until it acquires a channel in the random access interval. In the MAP, the transceiver is switched to the TX state according to the transmission time granted by the hub. Similarly, the node switches the transceiver to the RX state in the downlink interval approved by the hub. In other cases, the node goes to the SLEEP state.

#### 6.1.4. Mobility Model

**Figure 11:**
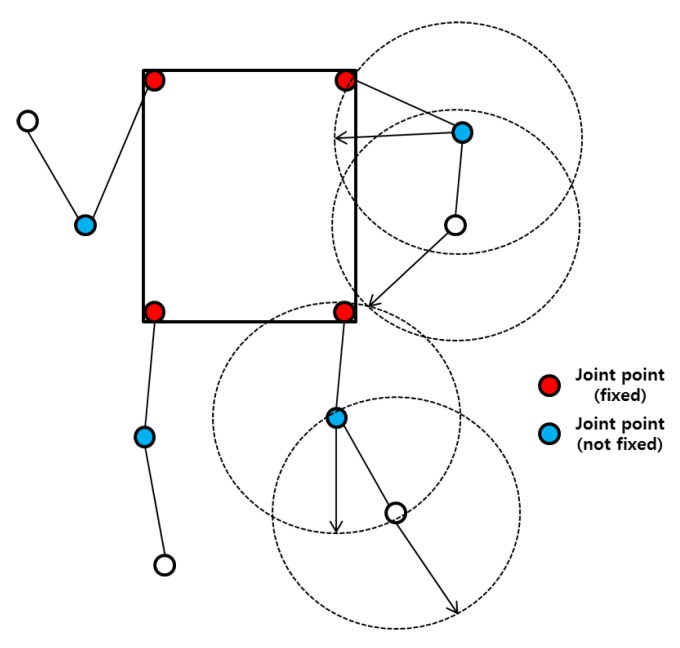
An example of the walking model.

The NS-3 simulator provides a variety of two-dimensional random mobility models, but they are not suitable for representing human motion. We provide an extra mobility model to represent realistic body movements. Here, **WbanMobilityModel** provides the three-dimensional vector position of a node, and models the node movement, including the velocity and direction. For example, the equation for expressing the rotation of an arm is as follows:

(4) $$position_{x}=rand\left(\right)\%2$$

(5) $$position_{y}=cos\left(-DegreesToRadians\left(D\right)\right)*C$$

(6) $$position_{z}=sin\left(-DegreesToRadians\left(D\right)\right)*C$$

where $position_{x}$ denotes the direction of movement, and rand() is a function that generates a random integer. The direction of movement is determined by a random value generated (i.e., 0 indicates the left direction, 1 indicates the right direction). The $position_{x}$ and $position_{y}$ represent the coordinates that represent the rotation of the node attached to the arm. Here, *D* stands for the current degree, and *C* is a constant value. Each node recursively moves to predefined coordinates according to the movement scenarios (e.g., walking, standing, running, and sitting). For example, we designate the joints to which the body’s limbs connect as fixed points. If we want to implement the walking mobility model, we connect the arms and legs to the joint points (fixed points) as shown in Figure 11. Each joint point is represented as a node, and the node at the fixed point is the root node. The child node rotates in a random direction based on the coordinates of the parent node.

### 6.2. Simulation Setup

The goals of the experimental studies are as follows; (i) we verify that the proposed simulation system meets the requirements for performance described in the IEEE 802.15.6 standard; and (ii) we verify that the proposed simulation system can support recent WBAN research topics regarding the dynamic resource allocation, inter-WBAN interference mitigation, and intra-WBAN routing. To achieve this goal, we build a simulation environment closest to the real WBAN environment and then construct specific simulation scenarios similar to the real health monitoring system. We evaluate the performance of the proposed system using NS-3 version 3.29. The simulation settings are divided into two categories, i.e., the network model and simulation configuration.

The network models used for the simulation are shown in Table 5. The supported frequency bands and transmission parameters follow NB PHY. The radio model in the NS-3 simulator assumes a flat channel frequency response. The modulation scheme for each frequency is not applied, but the error rate model is used based on the differential phase shift keying (DPSK) modulation with the highest data rate (971.4 kbps). The CSMA/CA is adopted as a random-access mechanism. The user priority for CSMA/CA is divided into eight levels, as shown in Table 4. I-Ack is adopted as an ACK policy. Among the three access modes, the beacon access mode with superframes, which includes three types of access phases (among EAP1, EAP2, RAP1, RAP2, MAP1, and MAP2), is adopted as an access mode. The simulation parameters are shown in Table 5. The maximum number of BANs is set to five, and each BAN consists of one hub and eight heterogeneous sensor nodes. The number of BANs is adjusted according to the simulation scenario. The length of an allocation slot is equal to pAllocationSlotMin+AllocationSlotLength(L)∗pAllocationSlotResolution, and the centralized guard time is set to pSIFS+pExtraIFS+mClockResolution.

### 6.3. Performance Metrics

To verify the performance of the proposed system, we use three principal performance metrics, i.e., throughput, average delay, and power consumption. We define the throughput as the amount of payload transmitted successfully within a given time period. The detailed equations for calculating other performance metrics are described in the following subsections.

#### 6.3.1. Average Delay

After sending a management type frame or data frame with an I-Ack policy to a node, and if a hub is expecting no more frames waiting for transmission or retransmission in the current allocation interval, the average delay (*D*) is defined as follows:

(7) $$D=T_{cu}+T_{pro}+T_{frame}$$

where $T_{cu}$ denotes the cumulative delay caused by the back-off time for a successfully transmitted frame, or the frame retransmission time, which includes the interframe space time (i.e., a node should wait a certain duration between the pSIFS and pExtraIFS before the retransmission of that frame). In addition, $T_{pro}$ denotes the propagation delay, and $T_{frame}$ denotes the total duration of a frame, which comprises the symbols for the PLCP preamble, PLCP header, and PSDU, and is calculated by [1] as follows:

(8) $$T_{frame}=T_{s}*\left(N_{preamble}+N_{header}*S_{header}+\frac{N_{total}}{log_{2}\left(M\right)}*S_{PSDU}\right)$$

where $T_{s}$, $S_{header}$, $S_{PSDU}$, and *M* are defined in the IEEE 802.15.6 standard, i.e., Tables 29 through 35, and $N_{preamble}$, $N_{header}$, and $N_{total}$ are defined in Sections 8.2, 8.3, and 8.4.4 in the IEEE 802.15.6 standard, respectively. In addition, $S_{header}$ and $S_{PSDU}$ refer to the spreading factor of the PLCP header and PSDU, and *M* denotes the cardinality of the constellation of a given modulation scheme.

#### 6.3.2. Power Consumption

The average power consumption (*E*) for a node is calculated as [22] follows:

(9) $$E=\left(\alpha -1\right)T_{cu}*Idle_{energy}+\left(\alpha \right)L*TX_{energy}+\left(\alpha *D\right)RX_{energy}+Sleep_{energy}$$

where α denotes the average number of back-off stages or frame retransmissions, and *L* denotes the length of the payload. In addition, $Idle_{energy}$, $TX_{energy}$, $RX_{energy}$, and $Sleep_{energy}$ denote the energy consumption in an idle state, RX state, RX state, and sleep state, respectively.

### 6.4. Verification Scenario 1: Heterogeneous Traffic Flow

According to the IEEE 802.15.6 standard, each BAN contains one hub and a range of nodes between 1 and 64. Each node collects different physical or chemical body data and transmits them to the hub based on the priorities of the body data. To send high-priority traffic, IEEE 802.15.6 MAC provides a priority-based random-access phase and managed access phase in the superframe. We analyze the performance of the proposed system under a scenario in which one hub transmits a beacon frame during each beacon period (superframe), except in inactive superframes. The node switches its state to inactive (IDLE) in EAP 1, RAP 1, EAP 2, or RAP 2, if it does not need to transmit a frame in the corresponding access phase. The EAP is used only to send emergency and critical traffic, and the RAP is used to send all types of traffic. MAP arranges the scheduled uplink, downlink, and bi-link allocation intervals, as well as unscheduled bi-link allocation intervals. Improvised polling allocation intervals are also optionally included in MAP to provide additional access periods without advanced notice. In this experiment, we aim to show the differences in performance according to three test cases to analyze the effects of MAP on the performance, such as the throughput and delay. Table 6 shows the beacon parameters used for each test case.

**Figure 12:**
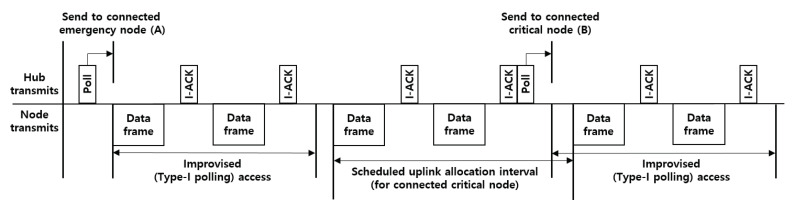
Frame transactions for improvised access in MAP.

Under this scenario, the number of BANs is set to one, and a hub and node are interconnected in a one-hop star topology. The MAC options (i.e., channel hopping and two-hop star topology extension) are not enabled. The other simulation parameters are shown in Table 5. After the hub assigns a connected_NID to an unconnected node through the connection assignment frame, it should provide scheduled allocation intervals and polled allocation intervals to the connected node in MAP. Note that the hub grants scheduled allocation intervals (Cases 2 and 3) and improvised Type-I polled allocation intervals (Case 3) only to emergency and critical nodes. As shown in Figure 12, the hub grants a Type-I polled allocation to the node, which starts at pSIFS and ends at the end of the allocation interval. By using this scenario, we verified that each node can achieve a differentiated QoS based on the traffic priorities.

#### Results and Discussion

In general, the throughput and delay vary considerably with different data rates (in this case, we set the data rate to 971.4 kbps) over the frequency bands. Except for the reason based on the PHY characteristics, there are two main reasons for the differences in the throughput and delay depending on the type of traffic. First, each node uniformly selects a random value from the interval ($CW_{min}$, $CW_{max}$) as a back-off counter during the contention access period (i.e., EAP1, RAP1, EAP2, and RAP2). That is, traffic with a low user priority has less chance of acquiring a channel during the contention access period. This is particularly true for BANs with large numbers of nodes. Second, a node with a high user priority (i.e., a node classified as an emergency or critical node) establishes an association with a hub and is assigned scheduled allocation intervals and improvised polling access intervals in MAP by the hub. That is, even if the emergency or critical node cannot acquire a channel during the contention access period, channel acquisition is guaranteed during the current superframe period.

**Figure 13:**
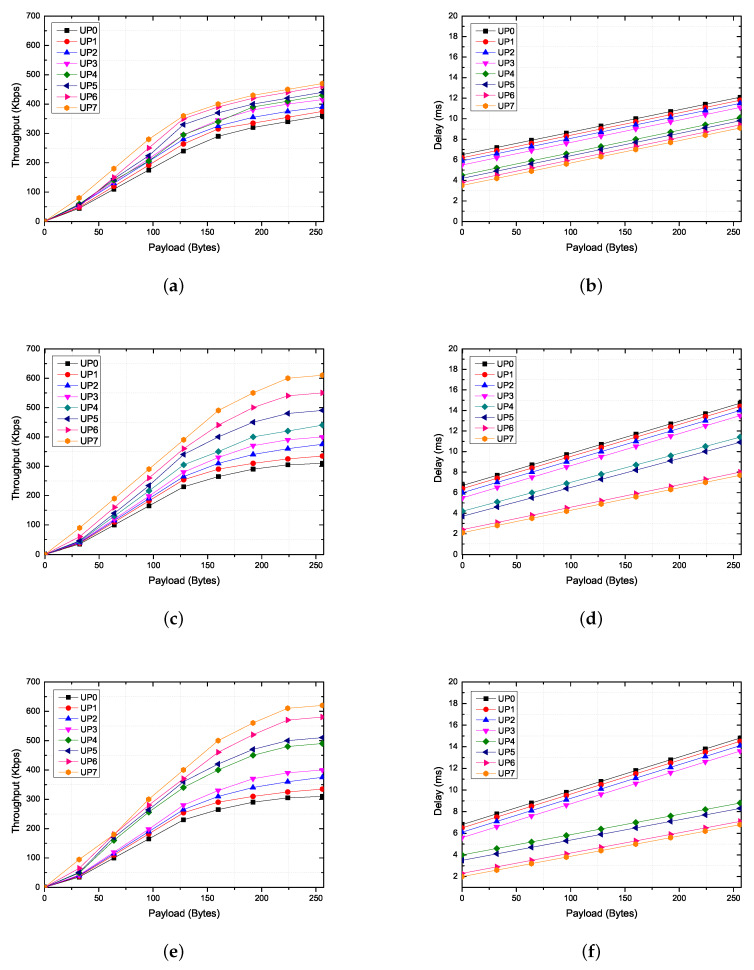
Performance comparison for different payloads under scenario 1: impact of varying payload on (**a**) throughput (Case 1), (**b**) average delay (Case 1), (**c**) throughput (Case 2), (**d**) average delay (Case 2), (**e**) throughput (Case 3), and (**f**) average delay (Case 3).

In case 1, only EAP 1, 2 and RAP 1, 2 exist in the superframe. Because nodes with higher UPs (i.e., $UP_{4}$ to $UP_{7}$) use EAP and RAP together, there are more opportunities to acquire channels than for nodes with lower UPs (i.e., $UP_{0}$ to $UP_{3}$). However, the difference in performance between each node is not great, as illustrated in Figure 13a,b. The reason is that all nodes acquire channels based on the CSMA/CA mechanism, even if the differentiated back-off counter is set according to user priority. The latency to acquire the channel increases as collision occurs, thereby reducing the overall throughput. That is, the contention-based channel access mechanism cannot satisfy the requirements of delay-sensitive medical applications because the allocation intervals are not guaranteed.

For case 2, MAP 1 and 2, including a scheduled uplink and downlink allocation interval, are added to the superframe. The nodes with higher UPs have higher transmission opportunities than nodes with lower UPs because the uplink allocation slots are preferentially allocated to the critical node through an association with the hub. For this reason, there is a large difference in throughput and delay between each node, as shown in Figure 13c,d. However, if the expected transmission time of the data including the guard time is larger than the remaining time slot, then the node will give up the current transmission. In addition, if the node no longer has data in the transmisssion queue, the remaining time slots can be wasted. Therefore, even if the scheduled allocation interval is long in the MAP, the performance will not be improved significantly.

For case 3, MAP 1 and 2, including a scheduled uplink, downlink, bi-link allocation interval, unscheduled bi-link interval, and improvised polling interval, are added to the superframe. By adding the type-I (immediate) polled allocation intervals to the MAP, unscheduled allocation intervals can be provided to critical nodes and wasted time slots can be reduced by adjusting the length of the scheduled uplink allocation interval. For example, owing to the UP-based scheduling policy, in case 2, the waste of uplink allocated slots may increase according to the data rates of the critical nodes (i.e., $UP_{6}$ and $UP_{7}$). However, in case 3, nodes with higher UPs can be assigned additional guaranteed time slots through a poll frame transmitted by the hub at the polling access interval. As illustrated in Figure 13e,f, the performance of a node with a higher UP (i.e., $UP_{4}$ to $UP_{7}$) is higher than that of a node with a lower UP (i.e., $UP_{0}$ to $UP_{3}$). In addition, the difference in performance between the emergency node (i.e., $UP_{4}$ and $UP_{5}$) and critical node (i.e., $UP_{6}$ and $UP_{7}$) is insignificant. The reason is that the wasted time slots can be reduced by adjusting the length of the scheduled uplink allocation interval, and additional allocation intervals are granted to the emergency node (i.e., $UP_{4}$ and $UP_{5}$) through improvised polling access.

### 6.5. Verification Scenario 2: Channel Sharing between Multi-WBANs

**Figure 14:**
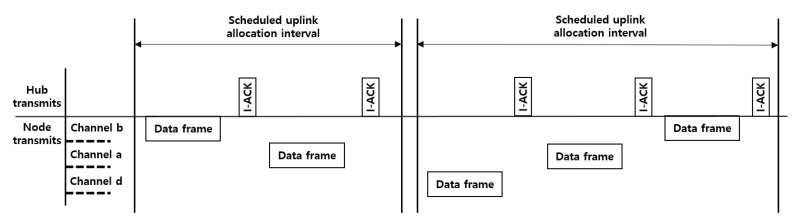
Frame transactions using channel hopping in MAP.

Most of the MAC protocols try to eliminate intra-WBAN interference. However, this is not realistic because most of the traffic volume is normal traffic, which can significantly reduce the throughput, and thus the overall performance of the BAN. Further, having smooth coexistence mechanisms with neighboring BANs is important to avoid inter-WBAN interference. In a multi-channel environment, the proposed system provides a channel-hopping mechanism for channel sharing between multiple WBANs. Channel hopping is recommended under dynamic mobility (e.g., patients moving fast in a hospital). A hub can enable a channel hopping option only if the PHY is set to NB PHY. As described before, a hub can change its operating channel periodically by including the channel hopping state and next channel hop fields in the beacon frame. The hub should choose a channel hopping sequence that is not being used by the neighboring hubs. The channel hopping sequence is set to the current state of a 16-bit maximum-length linear feedback shift register (LFSR) as specified in Section 6.13.2 of the IEEE 802.15.6 standard. As illustrated in Figure 14, the node may hop to new channels periodically based on the channel-hopping sequence. Note that the channel hopping does not require any message exchange between neighboring hubs.

Under this scenario, we configure a system of multiple WBANs, where the transmission ranges of nearby WBANs overlap each other, causing inter-network interference. The maximum number of neighboring BANs is set to five, and a hub and a node are interconnected in a one-hop star topology. In addition, the number of operating channels is set to 16. The other simulation parameters are the same as in scenario 1 (case 2). Using this scenario, we verify that the interference between multiple WBANs can be mitigated in the proposed system.

#### Results and Discussion

In addition to the main causes of the performance degradation of the throughput and delay, as described before, the additional performance reduction factor is channel interference by the neighboring BANs. The local BANs using the same frequency band cause significant channel interference. To mitigate this, each local BAN uses channel hopping through the beacon frame after associating between the hub and nodes.

**Figure 15:**
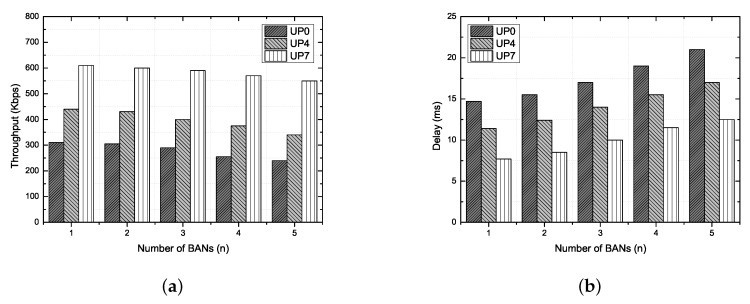
Performance comparison for different numbers of BANs in scenario 2: Impact of varying numbers of BANs on the (**a**) throughput and (**b**) average delay.

If channel hopping is currently enabled, the hub includes in its connection assignment frames a channel hopping field and ordering IE field indicating the selected channels. As shown in Figure 15a,b, even if the number of BANs increases, the performance of the throughput and delay does not significantly decrease. As the reason for this, the hub will hop to another channel after staying in the current channel for numerous superframe periods as communicated to the associated nodes. The hub generates a channel-hopping sequence based on the current channel state and number of coexisting BANs. The current version of the proposed simulation system simply chooses a channel hopping sequence that is not being used by its neighbor BANs. However, if there are more neighboring BANs than the number of available channels, collisions will occur. In addition, the hub needs to calculate a new channel hopping sequence and propagate the changed information to the node via the connection assignment frame, thereby the control overhead also increases. To overcome the limitation of the channel hopping mechanism, the hub can optimize the channel hopping sequence by utilizing historical data on the channel usage of the neighboring BANs. One of the solutions to solve the problem of the channel hopping mechanism is presented in Section 7. In conclusion, because the number of retransmissions from channel interference does not increase significantly, the QoS of traffic with a higher user priority can be guaranteed. Moreover, the contention for channel acquisition is applied independently for each BAN because the operating channels are no longer shared between coexisting BANs in the random access phase. As a result, normal traffic with a lower user priority can also maintain its performance.

### 6.6. Verification Scenario 3: Two-Hop Star Topology Extension

**Figure 16:**
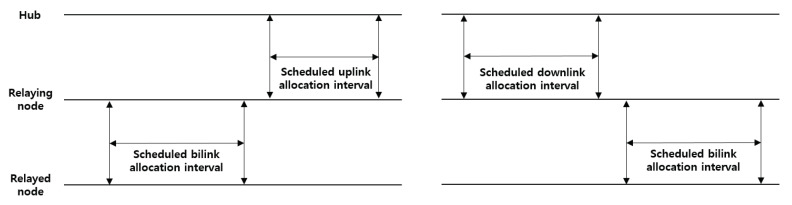
Two-hop scheduled allocations in MAP.

In a one-hop star topology, frame exchanges between the hub and nodes occur directly. However, the nodes far from the hub (e.g., nodes attached to the arms and legs) should use a large amount of transmission power, thereby compromising the lifetime of the network. However, in a two-hop star topology, a hub and node can exchange frames optionally through a relay-capable node. In addition, a two-hop star topology extension is a prerequisite supporting the routing protocol studied in a WBAN. To send a frame through the relaying node to the hub, as illustrated in Figure 7, the relayed node sends an encapsulating “X” frame to the relaying node, wherein the recipient ID field of the MAC header is set to the NID of the relaying node, and the relay field of the MAC header is set to 1. The frame payload is set to the encapsulated “X” frame. After receiving the encapsulating “X” frame from the relayed node, the relaying node forwards the frame to the hub in the same way as a one-hop star topology. To obtain scheduled allocation intervals for a two-hop extension, the relayed node should send an encapsulated connection request frame through the relaying node to the hub, as specified in Section 6.10.5 of the IEEE 802.15.6 standard. After the association between the relayed node and hub is completed, the relaying node and relayed node use their scheduled allocation intervals, as shown in Figure 16.

Under this scenario, the number of BANs is set to one, and a hub and several nodes are interconnected in a two-hop star topology. We set four normal nodes as relayed nodes and four emergency and critical nodes as relaying nodes. Unlike the previous scenarios, normal nodes are also assigned scheduled allocation intervals in MAP. The TX power of a relayed node is adaptively adjusted. The other simulation parameters are the same as in scenario 1 (case 2). Using this scenario, we verify the impact of the two-hop star topology extension on the overall power consumption.

#### Results and Discussion

**Figure 17:**
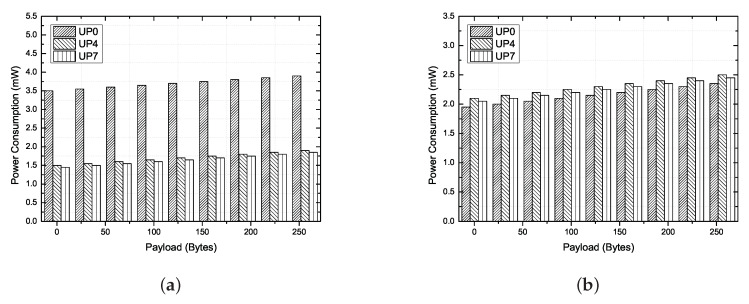
Performance comparison for different payloads in scenario 3: Impact of varying payloads on average power consumption in the (**a**) one- and (**b**) two-hop star topologies.

For energy saving, inactive or sleep mode and two-hop star topology extension mode can be applied to the proposed system. If a frame is pending in the buffer, the node sends the frame immediately to the hub during either the random-access phase or managed access phase. If the node has no frames to send to the hub, it may enter inactive mode. Moreover, the node goes to sleep during the inactivated superframe period. For all reasons above, the beacon mode with superframes is the best way to optimize the energy consumption in a one-hop star topology. However, a node located relatively far from the hub should consume a large amount of TX power. Figure 17a shows that $UP_{0}$ has the highest power consumption. The reason for this is that the nodes with a lower user priority use a large amount of TX power because they are mainly attached to the arms and legs. A node with a lower user priority accelerates an imbalance in power consumption because a retransmission is likely to occur between a hub and the node itself owing to a body shadowing. Figure 17b shows the energy consumption in a two-hop star topology extension mode. Here, $UP_{4}$ and $UP_{7}$ attached near the hub forward the “X” frame transmitted by $UP_{0}$ to the hub, and thus the power consumption is slightly increased, although the overall power consumption can be balanced.

### 6.7. Overall Discussion

By conducting comprehensive experimental studies, we demonstrate that the performance requirements of various medical sensors can be satisfied by thoroughly implementing the specifications described in the IEEE 802.15.6 standard. Unlike the existing WBAN simulation systems that support only contention-based random access, our simulation system supports heterogeneous traffic flow by implementing the MAP specified in the IEEE 802.15.6 standard. The MAP is essential to implement a connection-oriented link allocation mechanism between a hub and a node. From the experimental results, we can see that the transmission scheduling mechanism in MAP has a significant impact on throughput and delay. Our simulation system provides two optional mechanisms specified in the IEEE 802.15.6 standard, such as a channel hopping mechanism and two-hop topology extension, which are essential for efficient resource management. By defining frame transaction procedures for these additional mechanisms, our simulation system can support inter-WBAN interference mitigation and intra-WBAN routing studies.

In conclusion, our simulation system can provide a practical simulation environment for building a health monitoring system, and support the latest WBAN research topics regarding the dynamic resource allocation/transmission scheduling, inter-WBAN interference mitigation, and intra-WBAN routing. The WBAN research domains in which the proposed simulation system can be used are introduced in the next section.

## 7. Future Works and Challenges

The proposed system was developed based on the IEEE 802.15.6 standard. The IEEE 802.15.6 standard has already been optimized for body area networks, but there are some factors that can improve the performance or additional constraints that need to be reflected in the MAC design. The following sections describe the characteristics that should be considered in the IEEE 802.15.6 MAC protocol and present future studies that will be reflected in the proposed system.

### 7.1. Dynamic Transmission Scheduling

The WBAN topology may completely change because of changes in the posture and movement even within a certain posture type. The WBAN also moves as a whole within an ambient network. Unpredictable movements of the human body and changes in posture can significantly degrade the network performance such as the energy consumption, reliability, and delay. Particularly in terms of the energy consumption, ultra-low-power communication is required when considering the movement of the body because it is difficult to recharge the battery for each node inserted inside the human body. That is, the transmission policy should be adaptively changed according to the body posture and user priority for efficient resource management [32,33,34]. Human posture is defined by four positions (i.e., standing, sitting, lying, and walking), and a path loss model is developed according to a channel fading that occurs during each posture. We plan to study a transmission scheduling policy that maximizes the performance (i.e., a high throughput, low bit-error rate, and low delay) for each state after defining the state according to the body posture.

### 7.2. Dynamic Power Control

The specific absorption rate (SAR) is the rate at which the human body absorbs radio frequency (RF) radiation. In the United States, the Food and Drug Administration has the authority over health-related regulations for RF energy and issues regulations for acceptable limits of RF energy from electronic devices. Significant amounts of RF energy can increase the body temperature and cause tissue damage, particularly to the eyes and testes, which are more vulnerable owing to their smaller blood flow than other parts of the body for dissipating heat. To reduce tissue heating, the transmission power of the radio can be limited. However, existing studies on dynamic transmission power control have not taken into account the bio-effects [35,36,37]. We plan to propose a transmission power control model regarding the effect caused by biosensors to the human body in both near-field and far-field communication using the SAR. In addition, we will study an algorithm that calculates the optimal power required for transmission when a channel fading is expected between the node and coordinator when transmitting critical data.

### 7.3. Priority-Aware Time Slotted Channel Hopping

In a multi-channel environment, the proposed system provides a channel-hopping mechanism for channel sharing between multiple WBANs. The existing channel-hopping mechanism can avoid collisions with adjacent BANs in frequency bands that provide high data rates or large numbers of channels. However, when numerous local BANs simultaneously use a low-frequency band, the existing resource-sharing mechanisms need to be changed. That is, the channel sharing policy should guarantee the use of the channel for a critical data transmission [38,39]. In the future, we will study a priority-aware time-slotted channel-hopping algorithm between multiple WBANs to support the QoS of critical data.

### 7.4. Security Mechanism for Inter-WBAN and Beyond-WBAN Communications

To provide reliable security services for intra-WBAN communications, the IEEE 802.15.6 standard specifies a security association between a hub and a node. For secure communication with authenticated nodes, the hub uses the AES-128 encryption algorithm. Unlike intra-WBAN, which has limited capacity and computing power, a higher level of security technology should be applied for inter-WBAN communications or beyond-WBAN communications. In order to handle the protected health information generated by medical applications, the authors [40] propose utilizing blockchain-based smart contracts to facilitate secure analysis and management of medical sensors. Similarly, the authors [41] propose a hierarchical trust networking architecture based on a permissioned blockchain. Also, the authors [42] employ a blockchain to construct heterogeneous mobile edge computing (MEC) systems. The combination of blockchain and distributed multi-domain networks can effectively accomplish the topology privacy protection of MEC systems. In the future, we will study blockchain-based secure communication protocols for inter-WBAN and beyond-WBAN communications.

## 8. Conclusions

To build a realistic WBAN simulation system, we proposed the use of modeling strategies for implementing the IEEE 802.15.6 MAC on an NS-3 simulator. Unlike the existing WBAN simulation systems, we implemented additional mechanisms such as association, connection-oriented link allocation, a channel-hopping mechanism for mitigating inter-WBAN interference, and a two-hop star topology extension. In addition, we evaluated the performance of the proposed simulation system under realistic WBAN scenarios such as a heterogeneous traffic flow, channel sharing between multi-WBANs, and a two-hop star network topology. The simulation results demonstrate that the complementary mechanisms and realistic WBAN scenarios make our simulation platform closer to that of a practical WBAN system. Finally, we presented the unique characteristics of a WBAN that need to be considered in the IEEE 802.15.6 standard. Although the IEEE 802.15.6 standard has already been optimized for a practical WBAN system, there are additional factors degrading the overall performance. In a future study, we will present a modeling strategy to improve the link allocation and resource management scheme of the IEEE 802.15.6 standard.

## Figures and Tables

**Table 1:** Comparison of wireless body area network (WBAN) simulation systems.

Simulation Platform	Channel Access Mechanism	Association (Connection Oriented Resource Allocation Mechanism)	Mutual Interference Mitigation (Channel Sharing Mechanism)	QoS Support (Heterogeneous Traffic Flow)	Routing Support (Two-Hop Topology Extension)
C [9,12]	CSMA/CA, GTS	N	N	N	N
C++ [13,27]	CSMA/CA, GTS	N	N	Y	N
OMNET++ [10]	CSMA/CA, GTS, Poll	N	N	N	N
OPNET [11,14,15]	CSMA/CA	N	Y	Y	N
CANet [16]	CSMA/CA	N	N	Y	N
MATLAB [28,29]	CSMA/CA	N	N	Y	N
NS-3 [17,18,19]	CSMA/CA	N	N	Y	N
NS-3 (Proposed)	CSMA/CA, GTS, Poll	Y	Y	Y	Y

**Table 2:** Narrowband (NB) physical layer (PHY) specification.

Frequency Band	Modulation	Symbol Rate ($R_{s}\mathrm{ksps}$)	Code Rate (n,k)	Spreading Factor (SF)
402–405 MHz	π/DPSK	187.5	(31,19) (63,51)	2 {2, 1, 1, 1}
420–450 MHz	GMSK	187.5	(31,19) {(63,51), (63,51), 1}	2 {2, 1, 1}
863–870 MHz	π/DPSK	250	(31,19) (63,51)	2 {2, 1, 1, 1}
902–928 MHz	π/DPSK	250	(31,19) (63,51)	2 {2, 1, 1, 1}
950–958 MHz	π/DPSK	250	(31,19) (63,51)	2 {2, 1, 1, 1}
2360–2400 MHz	π/DPSK	600	(31,19) (63,51)	4 {4, 2, 1, 1}
2400–2483.5 MHz	π/DPSK	600	(31,19) (63,51)	4 {4, 2, 1, 1}

**Table 3:** Average gain for on-body channel model.

Node Pairs	Gain (dB)
Head to belt	−48.2
Head to wrist	−53.9
Belt to wrist	−50.0

**Table 4:** Bounds for CSMA/CA.

User Priority	${\mathrm{CW}}_{\min}$	${\mathrm{CW}}_{\max}$
0	16	64
1	16	32
2	8	32
3	8	16
4	4	16
5	4	8
6	2	8
7	1	4

**Table 5:** Simulation settings.

Network Model	Value
PHY	Narrowband PHY
Frequency band/Number of channels	2400–2483.5 MHz/16
Modulation/Symbol rate/Data rate	DPSK/600 Ksps/971.4 kbps
Noise	Additive white Gaussian noise (AWGN)
Propagation loss	Body shadowing and position-based
MAC	IEEE 802.15.6 (Beacon with superframes)
MAC options	Two-hop star topology extension/channel hopping/I-Ack policy
Mobility	Human mobility (walking, stand, sitting, etc.)
**Simulation Parameter**	**Value**
Number of BANs	1 to 5
Number of hubs	1
Number of nodes	8
Node (traffic) type	Normal (UP = 0, 1, 2, 3), emergency (UP = 4, 5), critical (UP = 6, 7)
$TX_{power}$/$RX_{sensitivity}$	−15 dBm/−83 dBm
$RX_{energy}$/$Idle_{energy}$/$Sleep_{energy}$	19 mA/0.4 mA/0.03 mA
Payload size	0 to 256 bytes
pAllocationSlotMin	500 us
pAllocationSlotResolution	500 us
AllocationSlotLength	1
Beacon length	240 ms
pSIFS/pMIFS/pExtraIFS	75 us/20 us/10 us
mClockResolution	4 us
mTimeout	30 us

**Table 6:** Beacon parameters.

**Case 1: Allocation Interval**	**Value (in Units of Allocation Slots)**
EAP 1	60
RAP 1	60
EAP 2	60
RAP 2	60
**Case 2: Allocation Interval**	**Value (in Units of Allocation Slots)**
EAP 1	20
RAP 1	20
EAP 2	20
RAP 2	20
Scheduled uplink interval (MAP 1,2)	80 (40 * 2)
Scheduled downlink interval (MAP 1,2)	80 (40 * 2)
**Case 3: Allocation Interval**	**Value (in Units of Allocation Slots)**
EAP 1	20
RAP 1	20
EAP 2	20
RAP 2	20
Scheduled uplink interval (MAP 1,2)	20 (10 * 2)
Improvised polled interval (MAP 1,2)	20 (10 * 2)
Unscheduled bi-link interval (MAP 1,2)	30 (15 * 2)
Scheduled downlink interval (MAP 1,2)	20 (10 * 2)
Improvised posted interval (MAP 1,2)	20 (10 * 2)
Improvised polled interval (MAP 1,2)	20 (10 * 2)
Scheduled bi-link interval (MAP 1,2)	30 (15 * 2)
